# Low-density lipoprotein particles in atherosclerosis

**DOI:** 10.3389/fphys.2022.931931

**Published:** 2022-08-30

**Authors:** Ya-Nan Qiao, Yan-Li Zou, Shou-Dong Guo

**Affiliations:** Innovative Drug Research Centre, School of Pharmacy, Institute of Lipid Metabolism and Atherosclerosis, Weifang Medical University, Weifang, China

**Keywords:** LDL particle, PCSK9, atherosclerosis, cardiovascular disease, PCSK9 inhibitor

## Abstract

Among the diseases causing human death, cardiovascular disease (CVD) remains number one according to the World Health Organization report in 2021. It is known that atherosclerosis is the pathological basis of CVD. Low-density lipoprotein (LDL) plays a pivotal role in the initiation and progression of atherosclerotic CVD (ASCVD). LDL cholesterol (LDL-C) is the traditional biological marker of LDL. However, large numbers of patients who have achieved the recommended LDL-C goals still have ASCVD risk. In multiple prospective studies, LDL particle (LDL-P) is reported to be more accurate in predicting CVD risk than LDL-C. LDL-Ps differ in size, density and chemical composition. Numerous clinical studies have proved that the atherogenic mechanisms of LDL-Ps are determined not only by LDL number and size but also by LDL modifications. Of note, small dense LDL (sdLDL) particles possess stronger atherogenic ability compared with large and intermediate LDL subfractions. Besides, oxidized LDL (ox-LDL) is another risk factor in atherosclerosis. Among the traditional lipid-lowering drugs, statins induce dramatic reductions in LDL-C and LDL-P to a lesser extend. Recently, proprotein convertase subtilsin/kexin type 9 inhibitors (PCSK9i) have been demonstrated to be effective in lowering the levels of LDL-C, LDL-P, as well as CVD events. In this article, we will make a short review of LDL metabolism, discuss the discordance between LDL-C and LDL-P, outline the atherogenic mechanisms of action of LDL by focusing on sdLDL and ox-LDL, summarize the methods used for measurement of LDL subclasses, and conclude the advances in LDL-lowering therapies using statins and PCSK9i.

## Introduction

According to the World Health Organization report in 2021, cardiovascular disease (CVD) causes 17.9 million deaths in 2019, representing 32% of total global deaths (WHO, 2021). The pathological basis of CVD is atherosclerosis, a disease of arteries causing myocardial infarction, stroke, and other complications (Libby et al., 2019). Atherosclerosis occurs primarily in the subendothelial space of the middle and large arteries, where blood flow is disturbed and/or bifurcated (Siasos et al., 2018; Kong et al., 2022). Accumulating evidence have demonstrated that many risk factors including hyperlipidemia, hypertension, oxidative stress, inflammation, endothelium dysfunction as well as high-calorie diet and unhealthy habits, such as smoking, contribute to the initiation and progression of atherosclerotic CVD (ASCVD) (Lee and Cooke, 2011). Epidemiological studies indicate that the elevated levels of low-density lipoprotein (LDL) cholesterol (LDL-C) are the major culprit in the development of atherosclerosis (Matsumoto et al., 2004; Khatana et al., 2020).

Although LDL-C is a well-accepted marker of LDL (Hero et al., 2016), epidemiologic studies and clinical trials using statins and other lipid-lowering drugs have demonstrated that LDL particle (LDL-P) is an alternative indicator and clinical target for treatment of dyslipidemia as well as ASCVD (Sniderman and Kwiterovich, 2013). Cholesterol, triglyceride (TG), and other components of LDL-P are not invariable, but differ greatly from person to person. For instance, lipids carried by LDL-P can be altered by lifestyle changes and lipid-modulatory therapies (Cromwell et al., 2007). In clinical practice, a large proportion of patients who have achieved the recommended LDL-C goals still suffer ASCVD. Therefore, LDL-C alone is not a necessary determinant for ASCVD, while LDL-P is found to be more predictive than any other parameters related to LDL (Kanonidou, 2021). LDL-Ps, the metabolism products of very low-density lipoprotein (VLDL) and intermediate density lipoprotein (IDL), are lipoprotein particles varying in size, components, and density (Maiolino et al., 2013; Ivanova et al., 2017). Of importance, not all LDL-Ps lead to atherosclerosis. Numerous clinical studies have demonstrated that the initiation and progression of atherosclerosis is determined by the number, size, and modification of LDL (Vekic et al., 2022). Particularly, small dense LDL (sdLDL) is widely distributed in the blood of patients with ASCVD and is sensitive to modifications, causing increased occurrence of atherosclerosis (Sekimoto et al., 2021). Moreover, many studies have clearly indicated that oxidized LDL (ox-LDL) and endothelium dysfunction are the main risk factors of atherosclerosis (Chisolm and Steinberg, 2000; Khatana et al., 2020). The measurements and atherogenic mechanisms of action of sdLDL and ox-LDL will be discussed in this review.

Apart from maintaining a healthy lifestyle, pharmaceutical interventions with hypolipidemic drugs, such as statins, are generally recommended for prevention and treatment of ASCVD (Gallego-Colon et al., 2020; Mach et al., 2020). In clinical practice, the effects of non-statin drugs on the outcome of CVD are inferior to those of statins (Visseren et al., 2021). As an add-on-statin therapy, proprotein convertase subtilsin/kexin type 9 (PCSK9) inhibitors (PCSK9i) have shown powerful effect on lowering LDL as well as ASCVD events (Cannon et al., 2015; Chaudhary et al., 2017). In this review, we will make a short review of LDL metabolism, discuss the discordance between LDL-C and LDL-P, outline the atherogenic metabolisms of action of sdLDL and ox-LDL, summarize the methods used for measurement of LDL subfractions, and conclude the current findings of LDL-lowering therapies using statins and PCSK9i. The literature in this article are searching results of the databases including PubMed and Web of Science primarily using “low-density lipoprotein particle or LDL-P” as the keyword.

## A brief review of LDL

LDL-P is composed of a lipid core that is primarily consisted of cholesterol, cholesterol ester (CE), and TG, and a shell that is consisted of phospholipids as well as dozens of proteins, such as the dominant apolipoprotein (Apo) B (Prassl and Laggner, 2008). The meaning of LDL varies based on the context. For instance, this term may refer to LDL-C, LDL-P number, or LDL Apo B (Rosenson et al., 2010). LDL-P varies in component, size, and density, while LDL-C specifically represents the cholesterol contained in the LDL-P (El Harchaoui et al., 2007).

### LDL metabolism

**FIGURE 1:**
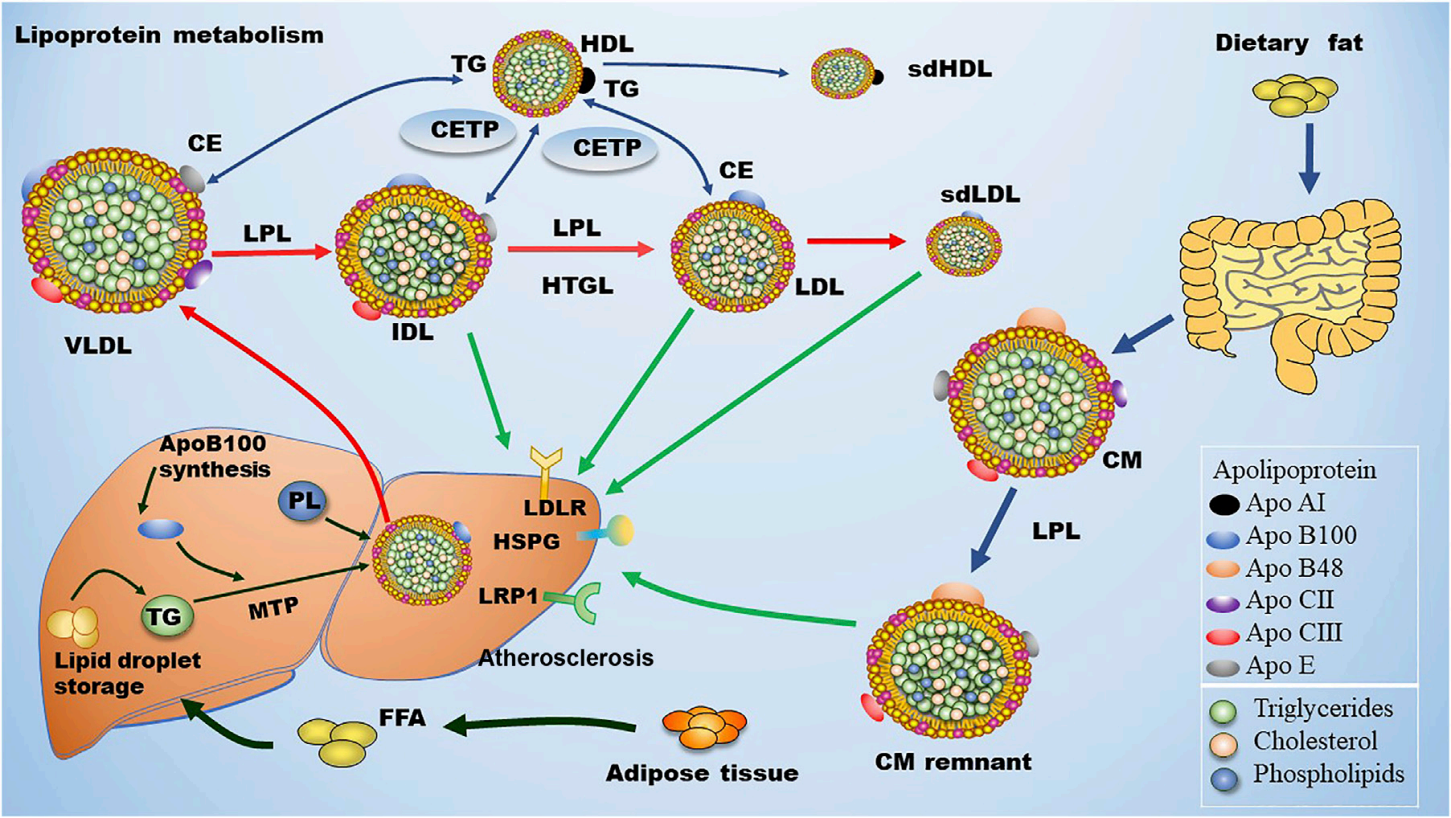
Metabolism of LDL. Dietary fat is degraded and then absorbed by intestinal cells for the assembly of chylomicron (CM), which is hydrolyzed by lipoprotein lipase (LPL) in circulation to produce chylomicron remnant (CMR). In the liver, apolipoprotein B (ApoB) 100 is critical for the generation of very low-density lipoprotein (VLDL). In blood, plasma VLDL is converted to intermediate low-density lipoprotein (IDL) and low-density lipoprotein (LDL) *via* hydrolysis of triglycerides (TGs) by LPL and hepatic lipase (HL). Of note, cholesteryl ester transfer protein (CETP) mediates the exchange of cholesterol ester (CE) and TG between high density lipoprotein (HDL) and Apo B-containing lipoprotein, leading to the production of small dense LDL (sdLDL) and small dense HDL (sdHDL), which are atherogenic factors. CMR, IDL, LDL, and sdLDL particles can be cleared by liver through LDL receptor (LDLR), LDLR-related protein 1 (LRP1), heparan sulfate proteoglycan (HSPG), and other potentially unknown receptors. MTP: microsomal triglyceride transfer protein.

**FIGURE 2:**
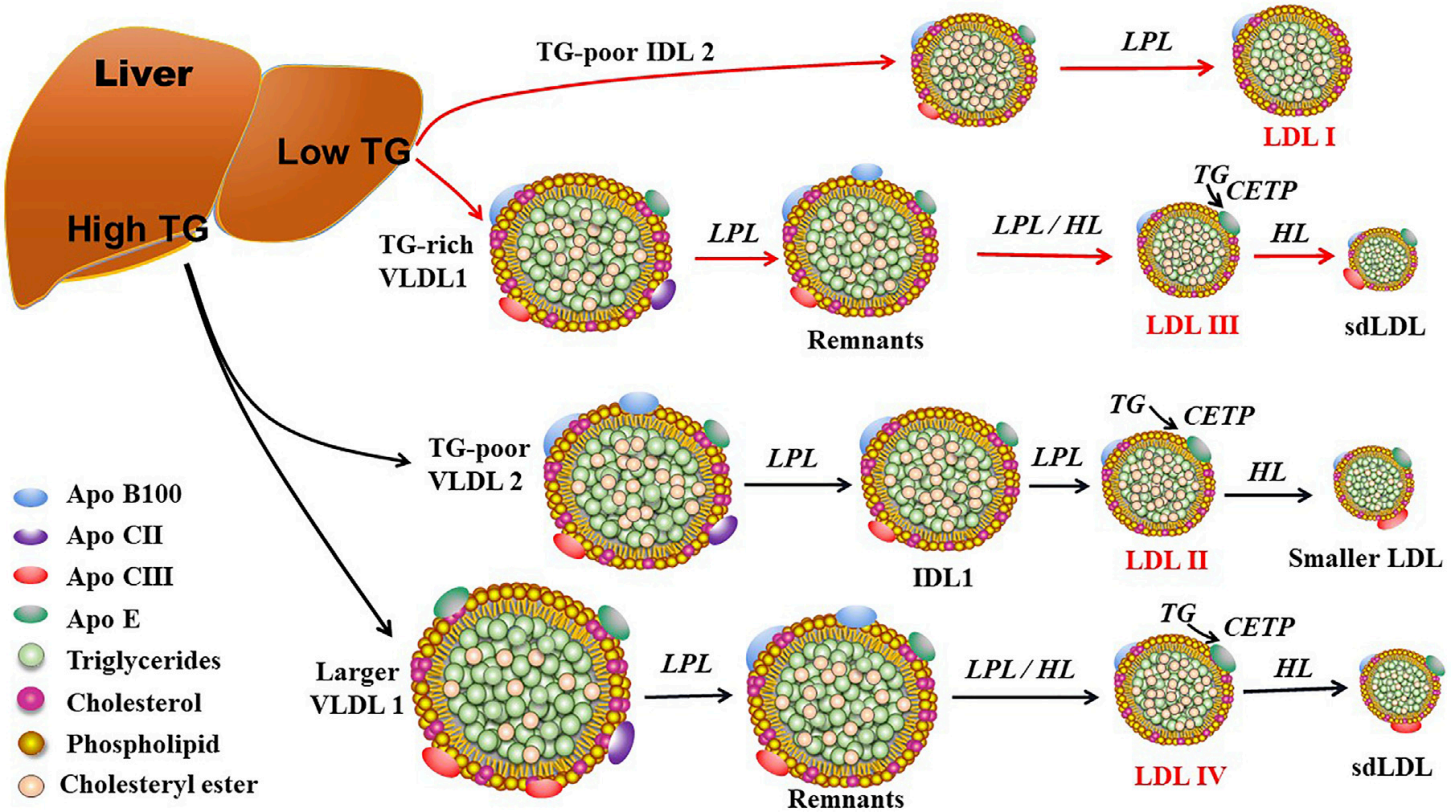
The proposed mechanisms for production of LDL subclasses I, II, III, and IV. LDL subclasses II, III, and IV can be further modulated by hepatic lipase (HL) and cholesteryl ester transfer protein (CETP). Of note, LDL subclasses III and IV are responsible for the production of small dense LDL (sdLDL). LPL: lipoprotein lipase; TG: triglyceride; VLDL, very low-density lipoprotein; IDL2: intermediate density lipoprotein 2; IDL1: intermediate low-density lipoprotein 1.

ApoB100 is the main structural protein of LDL, and there is only one ApoB100 molecule in each LDL-P (Young, 1990). ApoB100 is mostly synthesized in the liver, where Apo B100 is assembled with TG and other lipids as well as proteins to produce TG-rich VLDL particles (Figure 1). In circulation, VLDL particles are converted to IDL and LDL particles by lipoprotein lipase (LPL) and hepatic lipase (HL), which hydrolyze TGs in the core of the ApoB-containing particles including VLDL and chylomicron. As for the origin of LDL subclasses, Berneis and Krauss (2002) proposed two pathways dependent on hepatic TG availability. In case of low TG availability, liver secretes TG-rich VLDL1 and TG-poor IDL2, which are converted to LDLIII and LDLI particles, respectively (Figure 2). In case of high TG availability, liver secretes larger TG-rich VLDL1 and TG-poor VLDL2 particles, which are converted to LDLIV and LDLII, respectively (Figure 2) (Berneis and Krauss, 2002). Of note, cholesteryl ester transfer protein (CETP) mediates lipids exchange (TG and CE) between ApoB-containing lipoproteins and high-density lipoprotein (HDL) particles, contributing to the production of CE-enriched IDL and LDL particles as well as distinct LDL subclasses (Berneis and Krauss, 2002; Hirayama and Miida, 2012; Ivanova et al., 2017; Liu et al., 2021a).

Approximately 40%–60% of the total LDLs in blood are cleared by hepatic LDL receptor (LDLR) through binding the ligand ApoB100 that is carried by LDLs. The rest LDLs in blood are cleared by either hepatic LDLR-related protein (LRP) and heparan sulfate proteoglycan (HPSG) or non-hepatic non-LDLRs that are located at the inner surface of blood vessels (Mehta and Shapiro, 2022). The metabolism of LDL is summarized in Figure 1. Of note, LDLR expression is down-regulated upon increased dietary saturated fat and elevated hepatic uptake of cholesterol through chylomicrons. On the contrary, LDLR expression is up-regulated due to reductions in dietary fat and hepatic uptake of cholesterol. Non-hepatic scavenger receptors predominantly located at macrophages are responsible for engulfing the residual LDLs that are not cleared by hepatic receptors. Upon stimulation, monocytes penetrate into the subendothelial space of the artery and turn into macrophages. Next, these macrophages uptake modified LDL particles and become foam cells, initiating the formation of atherosclerotic plaques (Moore et al., 2013). ApoE serves as the ligand for hepatic clearance of ApoB-containing lipoproteins, especially chylomicron, from the blood except for LDL *via* interacting with ApoE receptors including the LRP1 and the VLDL receptor, which are not regulated by cellular cholesterol (Marais, 2019).

### LDL-C and LDL-P discordance

Numerous epidemiological studies and clinical trials have confirmed that LDL is a clinical target for treatment of ASCVD (Jeyarajah et al., 2007). In general, LDL levels are indirectly quantified by measurement of LDL-C, the content of cholesterol packaged in LDL-Ps. However, the content of cholesterol and other components in LDL-Ps differ from person to person and change over time due to alterations of lifestyle as well as drug intervention. Many proteins and enzymes can modify the size and components, especially the content of TC and TG, of LDL particles in circulation (Matyus et al., 2014). Of note, the expression of the genes and proteins involved in lipoprotein metabolism also varies among individuals. The above variable factors lead to the fact that LDL-C cannot represent the number of LDL-P at most of the situations.

When LDL-Ps are cholesterol-enriched, the levels of LDL-P number and ApoB will be overestimated by LDL-C levels and vice versa. For instance, subjects with hypertriglyceridemia have increased numbers of sdLDL particles that are relatively poor in cholesterol and CE and rich in TG compared with subjects with normal TG. Under the circumstances, LDL-C levels are sure to underestimate LDL-P numbers as well as Apo B100 levels (Fernández-Cidón et al., 2020). Similarly, LDL-C levels of subjects with more CE-enriched LDL-Ps, are sure to overestimate the number of LDL-P and the level of ApoB100 compared to those with normal or low levels of LDL-CE. The importance of LDL-Ps or ApoB in coronary artery disease (CAD) has been vigorously discussed for over 40 years (Sniderman et al., 2019; Sniderman et al., 2022). Dr. Sniderman has devoted his whole clinical and research career since 1973 on the importance of ApoB or LDL-Ps be the parameter to determine the severity of CAD. He is the first person to point out LDL-Ps per se, but not LDL-C as the determinate of CAD. Now, in 2022 the debate is over as written by Allan D. Sniderman et al. (2022).

Studies have indicated that the level of ApoB100 is a superior indicator of CVD risk compared with LDL-C (Sniderman et al., 2012; Sniderman et al., 2019). Each LDL-P consists of one molecule of ApoB100 (Young, 1990). Thus, the level of ApoB100 is well-correlated with the number of LDL-P. Alternatively, LDL-P level is a more convincing predictor of CVD risk compared with other parameters including LDL-C. LDL-P number, size, and modification are all important risk factors for atherosclerosis. The risk of atherosclerosis is aggravated when both the number and size of LDL-P are abnormal (Allaire et al., 2017). LDL-Ps with small size are easier to penetrate the vascular subendothelial space, where they are engulfed by macrophages, accelerating the formation of foam cells. Of note, the relationship between LDL-P size and atherosclerosis risk is affected by LDL-P number. LDL size becomes a non-causal risk factor for coronary heart disease (CHD) when LDL-P number is normal (Allaire et al., 2017). There are certain circumstances, such as inflammation and infections, cause the modification of native LDL. These modified LDLs also induce the formation of foam cells through scavenger receptor-mediated endocytosis (Rafieian-Kopaei et al., 2014). Collectively, LDL-P number, size, and modification are interconnected and contribute to the development of atherosclerosis together.

## Detection of LDL subclasses

**FIGURE 3:**
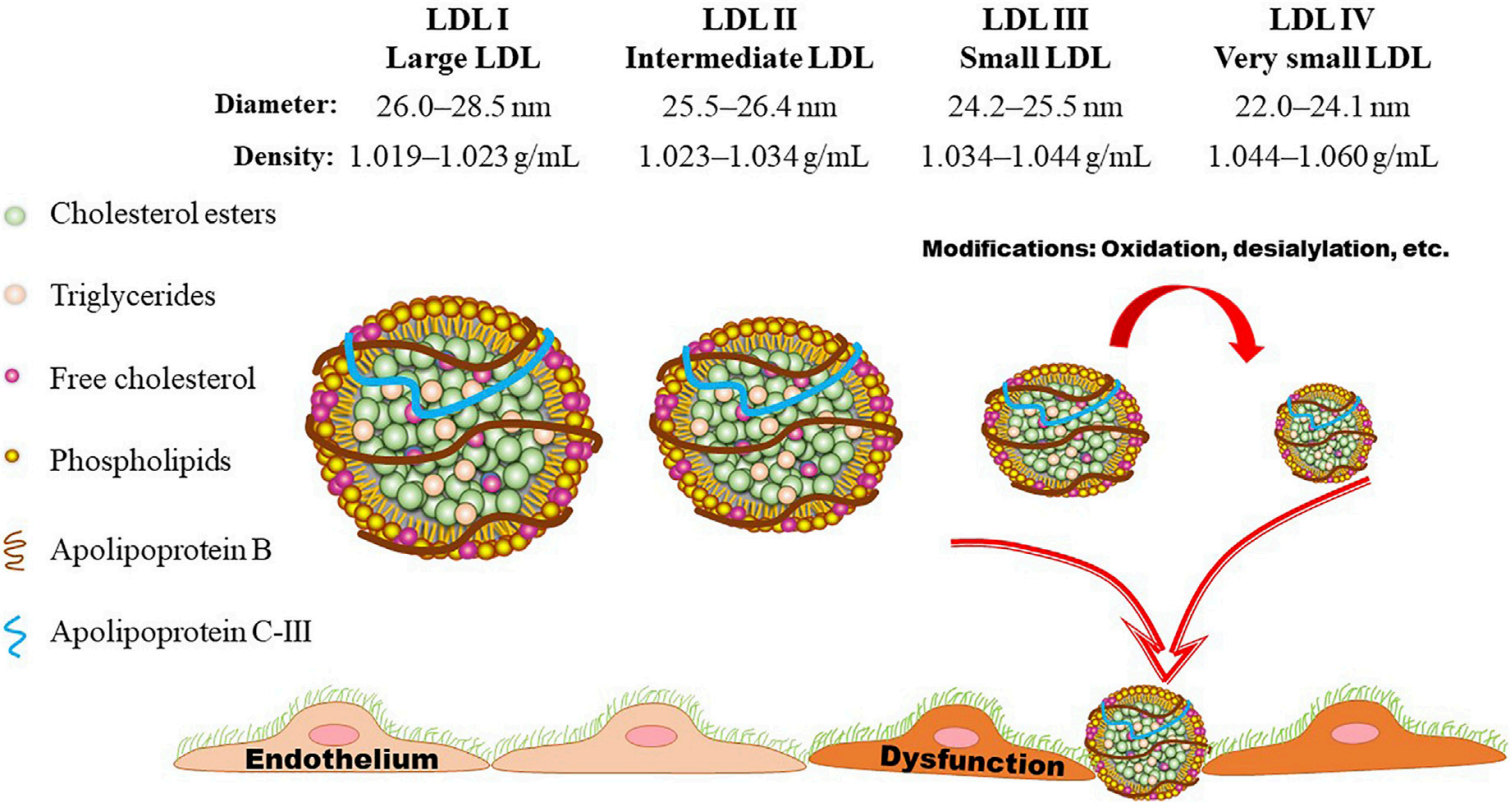
LDL subclasses. Based on the flotation rate, LDL particles can be divided into four subclasses by means of ultracentrifugation. Gradient gel electrophoresis is another commonly used method for separation of LDL subclasses according to the size, shape, and electric charge. LDL particles are generally divided into three or four subclasses according to their size (diameter). Presently, LDL III and LDL IV are generally assigned as small dense LDL (sdLDL), which is lack of anti-oxidant component and easily to be oxidized, initiating the onset and progression of atherosclerotic cardiovascular disease.

As shown in Figures 2, 3, LDL-Ps can be divided into three or four subclasses. The size (diameter) of large (LDL I) and intermediate (LDL II) LDL ranges from 26.0 to 28.5 nm and from 25.5 to 26.4 nm, respectively, while the size of sdLDL (LDL III and LDL IV) is generally less than 25.5 nm on average (Berneis et al., 2005; Hirayama and Miida, 2012). LDL-Ps can be divided into different subclasses according to their density, size, electric charge as well as protein composition. Based on their physical and chemical properties as listed above, LDL particles can be separated by different laboratory methods including ultracentrifugation, gradient gel electrophoresis (GGE), high-performance liquid chromatography (HPLC), as well as nuclear magnetic resonance (NMR). In general, results of LDL subclasses obtained using different methods cannot be compared directly due to the distinct characteristics including size and charge of these particles originated from the different classification principles.

### Ultracentrifugation

As shown in Figure 3, the density of LDL-Ps ranges from 1.019 to 1.060 g/ml. Ultracentrifugation is one of the mostly used method for separation of LDL-Ps according to their density (Ivanova et al., 2017). However, the density ranges of LDL subclasses have been designated differently by distinct groups (Teng et al., 1983). Differing from the values shown in Figure 3, the density range of LDL IV is also designated as 1.060–1.063 g/ml (Hirayama and Miida, 2012; Kanonidou, 2021), and the density range of LDL I and LDL III are also defined as 1.016–1.028 mg/ml and 1.028–1.043 mg/ml, respectively (Ivanova et al., 2017). Of note, the traditional gradient ultracentrifugation is time-consuming, while the vertical auto profile technique can identify four LDL subclasses simultaneously in less than an hour (Kulkarni, 2007). It is worth noting that the density of the obtained LDL subclasses varies mildly among different ultracentrifugation methods. For example, the density of the LDL subfractions obtained by iodixanol gradient ultracentrifugation is lower than that separated by traditional salt gradient ultracentrifugation (Davies et al., 2003; Yee et al., 2008). Although ultracentrifugation can separate LDL with high resolution, this method has low throughput and may cause overlap of LDL subclasses as well as destructive alterations due to the centrifugal forces as recently summarized by Kanonidou (Kanonidou, 2021).

### Gradient gel electrophoresis

GGE enables the prepared electrophoretic gel to form pore gradients from large to small, so that each component in the sample can pass through the gel with a decreasing pore size during the electrophoresis process, in order to obtain better separation (Kanonidou, 2021). GGE is widely used for separation of LDL subfractions based on size, shape, and electric charge. In this method, a 2%–16% sodium dodecyl sulfate-polyacrylamide gradient (SDS-PAGE) gel is generally used to separation LDL-Ps under non-denaturing conditions (Ensign et al., 2006). According to the peak diameter, LDL subclasses are separated into four subclasses including LDL I (26.0–28.5 nm), LDL II (25.5–26.4 nm), LDL III (24.2–25.5 nm), and LDL IV (22.0–24.1 nm) based on the calibration curve made by the standards that run in parallel with samples (Ivanova et al., 2017; Kanonidou, 2021). Furthermore, the LDL-Ps can be defined as pattern A (large and intermediate, > 25.5) and pattern B (small and very small LDL, ≤ 25.5 nm) (Ivanova et al., 2017). Similar to ultracentrifugation, GGE is also time-consuming and has a low throughput. Furthermore, this method needs standards for calibration. The polyacrylamide gel tube electrophoresis method is better for separation of LDL-Ps than the traditional GGE method. With the help of specific software, LDL-Ps can be separated into seven subclasses within 1 hour (Hirany et al., 2003).

Two-dimensional gel electrophoresis (2D-GE) is also used to separate LDL particles. In general, 300–500 μg of proteins are first isoelectric focused on immobilised gradient strips (pH 3–10). After equilibration, the strips are subjected to 2D-GE on a gradient SDS-PAGE gel (such as 8%–16%) (Sun et al., 2010; Ljunggren et al., 2019). This method can identify the alterations of proteins carried by LDL-Ps, even modifications of these proteins, in combination with MALDI mass spectrometry, thereby providing another way for examination of changes associated with ASCVD (Karlsson et al., 2005; Ke et al., 2020).

### Nuclear magnetic resonance

In the ^1^H-NMR spectrum, the terminal methyl protons of lipids under different chemical environments may exhibit distinct chemical shifts, which can be used to determine the LDL subclasses by comparation with the data documented in the libraries (Jeyarajah et al., 2007). According to the ^1^H-NMR data, the concentration of the LDL subclasses can be calculated as well as their size and lipid mass (Aru et al., 2017; Kanonidou, 2021). However, the LDL-P size measured by NMR (18.0–20.5 nm) is smaller than that determined by GGE. Therefore, the data obtained by different methods cannot be directly compared with each other (Kanonidou, 2021). Compared to ultracentrifugation and GGE, NMR can analyze samples within minutes without inducing destructive alterations. Based on the standardized protocols, the variation of NMR data performed by different labs is far less than 1% (Centelles et al., 2017). Therefore, NMR method is time-saving and reproducible and has the characteristic of high throughput.

### Fast protein liquid chromatography

Fast protein liquid chromatography (FPLC) method for detection of lipoprotein subclasses including LDL subclasses have been reviewed by Okazaki and Yamashita (Okazaki and Yamashita, 2016) and Kanonidou (Kanonidou, 2021). In combination with gel permeation columns and post-column reactions, this method can detect LDL-P size, LDL-P number as well as the levels of cholesterol and TG at a single run (Okazaki and Yamashita, 2016). Based on the previous report, FPLC method is feasible and straightforward.

### Clinical chemistry methods

Some commercially available kits and reference reagents have been developed and released by different companies, such as Quest and LabCorp in the United States. Traditional lipid testing measures the amount of LDL-C present in the blood, but it does not evaluate the number of LDL-P. LDL-P is often used to get a more accurate measure of LDL due to the variability of cholesterol content within a given LDL. Studies have shown that LDL-P more accurately predicts risk of CVD than LDL-C (Mora et al., 2014; Sniderman et al., 2022). These products have the characteristic of high throughput and have been used for common clinical assays in some countries.

Quest diagnostics released a series of advanced testing lipid panels, which go beyond standard lipid panels to assess lipoprotein and apolipoprotein risk factors (https://www.questdiagnostics.com/healthcare-professionals/about-our tests/cardiovascular/advanced-lipid). Quest provides advanced lipid testing including LDL-P number, small and medium LDL, LDL pattern, LDL peak size, and pholipase A2 (Ajala et al., 2020; Farukhi et al., 2020; Dugani et al., 2021). LabCorp also released several advanced testing lipid panels to assess the risk of developing CVD and monitor the treatment of unhealthy lipid levels (https://www.labcorp.com/help/patient-test-info/lipid-panel). These methods include LDL-P, ApoB, ApoA-I, lipoprotein (a), HDL particles, and cardiac risk assessment, which may provide deeper insights into the residual risk of patients with CVD (Ajala et al., 2020; Siddiqui et al., 2020). Furthermore, these companies provide methods for NMR analysis, such as the Lipo-Profile-3 algorithm (LabCorp) (Porter Starr et al., 2019; Lo et al., 2022). It is thought that these values may more accurately reflect heart disease risk in certain people.

LDL-P testing evaluates LDL particles according to their concentration in the blood. It may provide useful information to further evaluate an individual’s CVD risk if one has a personal or family history of heart disease at a young age, especially if one’s TC and LDL-C values are not significantly elevated. Furthermore, Cardio IQ^®^ is one of advanced cardiovascular testing methods from Quest, which can uncover hidden risk for heart attack and stroke help and improve the management of cardiovascular patients by testing emerging biomarkers such as LDL-P number and subclasses using ion mobility method. The ion mobility method for measuring lipoprotein subfraction concentrations is unique in its capability of directly determining concentrations of the full lipoprotein spectrum (VLDL, IDL, LDL, and HDL) independent of the particles’ cholesterol composition (Kanonidou, 2021). Ion mobility determines particle number after separating lipoprotein particles by size using gas-phase electrophoresis and directly counting the size-separated particles (Caulfield et al., 2008; Mora et al., 2015). Ion mobility method is commercially available from companies such as Quest.

### Other methods

The electrospray differential mobility analysis is also used for quantification of non-HDL particles. However, the machine needs to be daily calibrated by the reference standards, whose nature and composition should be close to the interested samples (Clouet Foraison et al., 2017). ApoB and non-HDL particles can also be detected by liquid-chromatography tandem mass spectrometry, but this method can’t provide information on lipoprotein subclasses at present (Delatour et al., 2018). Additionally, the research interest in the electrochemical immunosensors for the LDL detection has been constantly growing. Aptamers including oligonucleotides or peptides have characteristics of high reproducibility and high throughput. However, these immunosensors cannot detect the LDL subclasses at present (Rudewicz-Kowalczyk and Grabowska, 2021).

## Atherogenic mechanisms of LDL

LDL behaves as a chief risk factor in the initiation and progression of ASCVD (Ivanova et al., 2017). Of note, the number, size, as well as modifications of LDL play pivotal roles in the development of atherosclerosis (Packard, 2006; Rizzo and Berneis, 2006). Herein, we focus on two kinds of specific LDLs, sdLDL and ox-LDL, which have great atherogenic effects.

### sdLDL in atherosclerosis

As shown in Figure 3, the size of LDL-Ps ranges from large to small, and sdLDL is generally designated as small and very small LDL subclasses (Rosenson et al., 2010). It has been demonstrated that sdLDL is more atherogenic than large and intermediate LDL subclasses (Nikolic et al., 2013; Ivanova et al., 2017). Elevated levels of sdLDL are linked to atherosclerosis in many conditions, such as hyperlipidemia, metabolic syndrome, diabetes, and other disorders (Toledo et al., 2006; Cali et al., 2007; Fukushima et al., 2011). For instance, the proportion of sdLDL can be used to predict the elevated intima-media thickness (IMT) as well as insulin resistance in patients with diabetes (Gerber et al., 2013). The size of LDL-Ps decreases as insulin resistance becomes more severe (Garvey et al., 2003; Bonilha et al., 2021). Of note, the elevated levels of carotid IMT and sdLDL are correlated with other well-known risk factors of CVD including sex, age, genetics, as well as unhealthy habits such as smoking. Furthermore, sdLDL cholesterol is reported to be a superior indicator of CVD risk compared to other risk factors as listed above (Shen et al., 2015). The convincing correlation between sdLDL cholesterol and CHD is established in a prospective study involving 11,419 participants (Hoogeveen et al., 2014). Another study also indicates that sdLDL cholesterol is a better predictor of CHD than LDL-C (Ivanova et al., 2017).

Compared with large and intermediate LDL subclasses, sdLDL particles are more atherogenic. The small size makes sdLDL easily penetrate into the subendothelial space, where they bind more avidly to the glycosaminoglycans and are engulfed by macrophages to promote the formation of atherosclerotic plaques (Sniderman et al., 2019). Compared to large LDL-P, the longer circulation time of sdLDL provides more chances for its penetration into the subendothelial space (Carmena et al., 2004; Ivanova et al., 2017). *In vitro*, sdLDL particles are easier to be engulfed by macrophages compared with larger and less dense LDL-Ps. The potential reasons are: 1) sdLDL is more sensitive to oxidation; 2) sdLDL has a stronger binding ability to proteoglycans located at the endothelium lining (Alsaweed, 2021). Furthermore, the elevated plasma levels of sdLDL are generally accompanied with reduced levels of HDL-C and Apo A-I, and elevated levels of TG and ApoB. It has been well-documented that HDL and Apo A-I are atheroprotective, while TG- and ApoB-containing lipoproteins are atherogenic (Diffenderfer and Schaefer, 2014; Zhang et al., 2022).

### Ox-LDL in atherosclerosis

The higher the amount of LDL being trapped in the subendothelium, the faster the atherosclerotic plaque evolves (Rosenson and Underberg, 2013; Liu et al., 2021b; Lin et al., 2021). It is worth noting that only after modifications, such as oxidation and desialylation, LDL particles become atherogenic. Of note, ox-LDL has been a major risk factor in atherosclerosis (Mitra et al., 2011; Ahmadi et al., 2021; Lin et al., 2021).

Reactive oxygen species (ROS) and reactive nitrogen species are important contributors of LDL oxidation. In the vessels, reactive species can be produced by nicotinamide adenine dinucleotide phosphate (NADPH) oxidase, xanthine oxidase, lipoxygenases, cyclooxygenase, myeloperoxidases, nitric oxide synthase, and uncoupled endothelial nitric oxide synthetase, dysfunctional mitochondria as well as metal ion catalysis (Kattoor et al., 2017; Malekmohammad et al., 2019). The subendothelial space is the presumed site of LDL oxidation (Matsuura et al., 2008). Lipids of LDL are the primary targets of reactive species. Lipid peroxidation includes peroxidation of phospholipid and CE at the polyunsaturated fatty acid moieties. This process generates more reactive aldehyde products and metabolites including malondialdehyde and 4-hydroxynoneal which are linked to ApoB, phosphatidylserine, and phosphatidylethanolamine, through building adduct with Schiff-base lysine residue (Niki and Noguchi, 1997; Khatana et al., 2020). In general, CE 18:2 hydroperoxide, CE 18:2 hydroxide, phosphatidylcholine hyderoperoxide, ketone, oxidized ApoB, oxidized sphingomyelin, and 7-ketocholesterol are major oxidized components of ox-LDL (Yoshida and Kisugi, 2010; Khatana et al., 2020). The mechanisms of action of LDL oxidation have been reviewed by distinct groups (Yoshida and Kisugi, 2010; Khatana et al., 2020; Koschinsky and Boffa., 2022). A recent *in vitro* study suggests that riboflavin-sensitized photooxidation is also a potential mechanism of LDL oxidation and this process is critical for the development of CVD (Yeo and Shahidi, 2021). However, the above novel mechanism needs to be verified *in vivo* in future.

Some antioxidants are found to counteract the oxidation process. The enzymatic antioxidants in the blood vessels are superoxide dismutase, glutathione peroxidase, catalase, paraoxonase, and thioredoxin reductase (Kattoor et al., 2017). The non-enzymatic antioxidants include glutathione, coenzyme Q/coenzyme QH2, uric acid, bilirubin, lipoic acid, Vitamin E, and Vitamin C (Siekmeier et al., 2007; Malekmohammad et al., 2019). These antioxidants exert important protective functions against oxidation. For instance, glutathione, a cofactor for glutathione peroxidase scavenges hydroxide, hypochlorous acid, and peroxinitrite, thus modulates atherosclerosis (Pastore and Piemonte, 2013). Coenzyme Q improves endothelial function by scavenging peroxyl radicals (Moss and Ramji, 2016). The mechanisms of action of these antioxidants have been well documented by Malekmohammad et al. (2019) and Kattoor et al. (2017).

Of importance, ox-LDL and macrophages are involved in the whole process of atherosclerosis from plaque initiation to plaque progression, rupture, or even regression (Liu et al., 2021a; Lin et al., 2021). Ox-LDL elicits atherosclerotic events right from their production in the subendothelium. Ox-LDL, *via* lectin-like ox-LDL receptor (LOX1) and other factors, activates endothelium for a number of events: adherence of LDL, monocytes, and platelets; secretion of chemokines and growth factors; production of ROS; impairing NO secretion; and so on. Scavenger receptors, CD36, and LOX1 assist the uptake of ox-LDL by monocyte-derived macrophages in the subendothelium (Kattoor et al., 2019). Growth factors mediate proliferation of smooth muscle cell and formation of extracellular matrix. Platelet adherence and accumulation are also, in part elicited by ox-LDL which result into a rupture prone thrombus (Kruth et al., 2002; Yoshida, 2010; Khatana et al., 2020; Liu et al., 2021b; Lin et al., 2021). The mechanisms of action of ox-LDL-induced atherosclerosis have been well-documented (Matsuura et al., 2008; Khatana et al., 2020; Jiang et al., 2022).

The plasma level of ox-LDL is positively associated with the severity of CVD, suggesting ox-LDL is a valuable biomarker of CVD (Ivanova et al., 2017). Presently, several immunological techniques with high sensitivity and reproducibility have been developed for measurement of ox-LDL (Kohno et al., 2000; Fang et al., 2002). The methods using antibodies that are specifically binds to the oxidized components of LDL are sure to improve the detection efficiency and accuracy of ox-LDL. However, these methods are different in principle and operation procedures. Controversial results may be obtained *via* distinct detection methods (Itabe and Ueda, 2007).

## Therapies targeting LDL-P

The 2022 American College of Cardiology (ACC)/American Heart Association (AHA)/American Heart Failure Society (HFSA) guidelines support the use of statins to prevent CVD events (Heidenreich et al., 2022). In clinical practice, “the lower the better” is still a widely accepted principle for lowering LDL-C. Except for statins, other hypolipidemic agents are also applied for treatment of dyslipidemias. Presently, the overall effect of non-statins is inferior to that of statins. However, PCSK9i show attractive lipid-lowering and anti-atherosclerotic effects in practice (Cannon et al., 2015; Chaudhary et al., 2017; Guo et al., 2020). As we reviewed recently, angiopoietin-like protein (ANGPTL), such as ANGPTL3, plays a key in regulation of both cholesterol and TG *via* inhibition of LPL (Zhang et al., 2022). The antisense oligonucleotide of ANGPTL3 has been applied to familial hypercholesterolemia and is now in phase 3 clinical trial (Graham et al., 2017). In the following, we will briefly review the hypolipidemic effects and mechanisms of action of statins and PCSK9i.

### Statin therapy

Mechanistically, statins primarily reduce the serum level of cholesterol by inhibiting the activity of HMG-CoA reductase. HMG-CoA reductase is the rate-limiting enzyme for cholesterol formation in the liver and other tissues. By inhibiting HMG-CoA reductase, statins reduce the hepatocyte cholesterol content, stimulate the expression of LDLR, and ultimately enhance removal of LDL-C from the circulation (Toth, 2010). Except for lowering LDL-C, recent studies suggest that statins can effectively reduce the number of LDL-P (Folse et al., 2014; Heidenreich et al., 2022). However, statins are more effective in reducing LDL-C than lowering LDL-P. Furthermore, hepatic LDLR is not effective in clearance of sdLDL compared to normal-sized LDL. Therefore, a proportion of patients treated with statins remain high levels of LDL-P due to the discordance between LDL-C and LDL-P (Folse et al., 2014). The inefficiency of statins in reduction of LDL-P may explain, at least in part, the residual CVD risk in patients who have achieved the recommended LDL-C goals (Wong et al., 2017). A series of study have suggested that statin therapy guided by reductions in LDL-P can further decrease the CVD risk compared with that guided by LDL-C alone in high-risk patients. Thus, LDL-P guided therapy is an effective way to reduce the residual CVD risk. Furthermore, statin therapy is reported to reduce the level of plasma ox-LDL in patients with different diseases such as acute ischemic stroke (Tsai et al., 2014; Mansouri et al., 2022). As reviewed recently, statins are found to modulate the nuclear factor erythroid two related factor 2/heme oxygenase-1 signaling pathway in different diseases (Mansouri et al., 2022).

**FIGURE 4:**
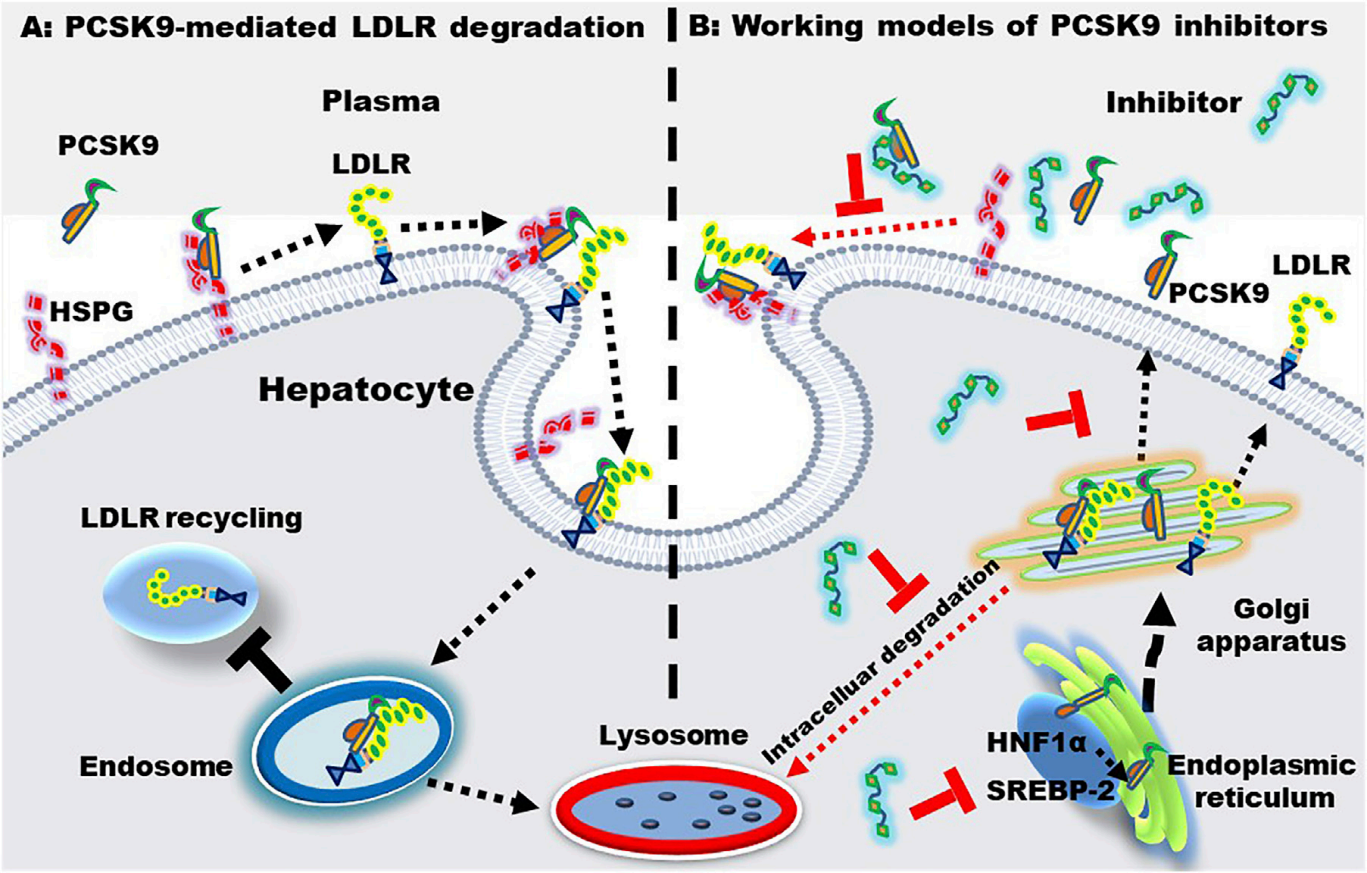
PCSK9-mediated LDLR degradation and the working models of PCSK9 inhibitors. **(A)** PCSK9-mediated LDLR degradation. Heparan sulfate proteoglycan (HSPG) located at the hepatic surface captures PCSK9, enhancing the binding between PCSK9 and LDLR. Upon binding, the PCSK9-LDLR complex is internalized and transferred to endosome, where PCSK9 binds LDLR with an even higher affinity. Finally, the complex is transported to the lysosome for degradation. **(B)** working models of PCSK9 inhibitors. PCSK9 inhibitors may inhibit PCSK9-LDLR interactions, PCSK9 production, transcription, and secretion. The PCSK9 expression is regulated by sterol regulatory binding protein-2 (SREBP-2) and hepatocyte nuclear factor 1α (HNF-1α).

Most LDL-Ps are cleared by LDLR that located at the surface of hepatocytes. Upon binding, LDL-P and LDLR are combined to form a complex that is endocytosed by hepatocytes. In general, the LDL and LDLR complex will separate in endosome and the released LDLR is transported back to the surface of hepatocytes to capture LDL-Ps again, further reducing the plasma LDL level. LDL-Ps, on the other hand, are degraded in lysosomes. These mechanisms of action of LDLR are summarized in Figure 4A. Of note, statins upregulate LDLR through sterol regulatory binding protein-2, which also enhance the expression of PCSK9. Accumulating evidence have demonstrated that PCSK9 induces the formation of LDLR-PCSK9 complex, leading to the degradation of LDLR in lysosomes, thereby reducing LDLR recycling and LDL-P clearance (Guo et al., 2020; Kong et al., 2022).

### PCSK9i

Statin intervention is associated with side effects as well as intolerability. Of importance, a proportion of patients with hyperlipidemias fail to meet the targeted LDL-C levels even using the maximum tolerated dose of statin (De Backer et al., 2019). Furthermore, clinical data have displayed that > 70% of patients with ASCVD fail to reach the recommended level of LDL-C < 70 mg/dl (Adhyaru and Jacobson, 2018). Of note, PCSK9 inhibition enhances the amount of hepatic LDLRs, leading to a substantial decrease of LDL-Ps in circulation. PCSK9i can be divided into three categories: 1) monoclonal antibodies or mimic antibody proteins which directly inhibit PCSK9 protein from binding to LDLR; 2) small interfering RNA (siRNA) and antisense oligonucleotides which inhibit PCSK9 production *via* gene silencing; 3) small molecules with multiple modulatory functions. The mechanisms of action of PCSK9i are shown in Figure 4B. Of importance, PCSK9i reduce the production of LDL-Ps, while statins do not change the production rate of LDL-Ps (Sniderman et al., 2017). As mentioned previously, statins increase the level of PCSK9 which reduces LDLR recycling and LDL-P clearance. Therefore, PCSK9i have different LDL-P-lowering effects and mechanisms compared to statins.

Besides, PCSK9i play a potential role in modulating the production of ox-LDL. Several studies demonstrated that elevated circulating PCSK9 directly enhances platelet activation, which induces CVD (Cammisotto et al., 2020; Qi et al., 2021). PCSK9i treatment inhibits platelet activation by modulating ox-LDL production and ox-LDL-mediated signaling pathway (Cammisotto et al., 2021). As reviewed recently, PCSK9 enhances the expression of NADPH oxidase and promotes the production of ROS and ox-LDL; PCSK9i can reduce oxidative stress *via* improving the activity of antioxidants such as glutathione peroxidase, superoxide dismutase, and catalase, thereby counteracting lipid peroxidation, the production of ox-LDL as well as cell damage (Cammisotto et al., 2022). Of note, pre-clinical trials have reported an independent association between plasma levels of PCSK9 and small LDL subfractions in patients with established CVD (Zhang et al., 2015; Lankin et al., 2018). A novel PCSK9 inhibitor RG7652 is reported to reduce both the small and large LDL-Ps, but the median percentage change is lower for the smaller LDL-Ps compared to the larger LDL-Ps (−43% vs. −81% from baseline), and 11 of the 45 patients showed an increase in the small LDL-Ps at the end of the study (Baruch et al., 2017). The PCSK9 monoclonal antibodies, alirocumab and evolocumab, are also reported to reduce larger LDL-Ps more powerfully than small LDL-Ps (Koren et al., 2015; Wu et al., 2017). To summarize, PCSK9 inhibitors seem to be effective at lowering all LDL subfractions, but with a trend towards a more efficient lowering of the larger LDL subfractions.

The development of PCSK9 monoclonal antibodies including evolocumab and alirocumab have been demonstrated to lower LDL-C levels as well as CVD risks (Hadjiphilippou and Ray, 2017; Schwartz et al., 2018). However, the biweekly or monthly subcutaneous injection of these antibodies has been a major concern for patient compliance. The siRNA, inclisiran, is developed to target and reduce the gene expression of PCSK9 by approximately 80% in all three Phase III ORION studies (Hardy et al., 2021; Henney et al., 2021). Inclisiran markedly reduces hepatic generation of PCSK9, causing a profound decrease in LDL-C level > 50% compared with control. Of note, this synthetic siRNA can maintain its pharmacological effects within half a year. However, whether inclisiran can reduce CVD outcomes is still being evaluated at present (ORION-4). Additionally, assessment of the long-term tolerability, efficacy, and safety of inclisiran needs to be continued based on a larger group of patients (Merćep et al., 2022). The gene therapy targeting PCSK9 has been reviewed recently by different research groups (Henney et al., 2021; Miname et al., 2021; Katzmann et al., 2022).

Cost-effective small-molecules with specific PCSK9 inhibition activity is an attractive research field. The advances of these inhibitors including derivatives of guanidine, berberine, piperidine, imidazole, and benzimidazole, have been well-documented recently by Ahamad et al. (2022). Of note, accumulating evidence have demonstrated that natural products are an important resource for discovery of PCSK9i. These natural products include berberine, lupin, quercetin, resveratrol, and others as reviewed by several groups (Ahamad et al., 2022; Waiz et al., 2022). Mechanistically, these small molecules may directly inhibit PCSK9-LDLR interactions, PCSK9 production, transcription, and secretion (Ahamad et al., 2022). Although some advances have been achieved, none of these small-molecule PCSK9i has been approved by the Food and Drug Administration of any country.

## Conclusion and future perspetive

Recent studies have demonstrated that LDL-P is a superior indicator of ASCVD risk than LDL-C. Of note, LDL-Ps are divided into various subclasses which vary in atherogenicity. SdLDL and ox-LDL are found to be more atherogenic compared with other LDL-Ps. However, study of the roles of these LDL subclasses, such as sdLDL, in the development of ASCVD is not easy because distinct methods may obtain different LDL subclasses with distinct physiochemical properties. At present, it is still early to determine which of the available methods is the most accurate and suitable for clinical use. Therefore, it is impelled to explore standard methods for preparation and evaluation of these LDL subclasses. Quest and LabCorp in the United states have established some commercially available methods for clinical assays with high throughput.

Statins are effective in reducing LDL-C level and even in decreasing LDL-P number. However, statin intervention is associated with side effects as well as intolerability. Furthermore, a large number of patients fail to reach the desirable LDL-C goals even with the maximum tolerated doses of statins. Furthermore, statin intervention enhances the levels of PCSK9, which accelerates LDLR degradation, causing the reduced ability of statins for lowering LDL-P number. Of note, PCSK9 monoclonal antibodies increase the expression of LDLR proteins, leading to profound reductions in plasma levels of LDL-P. Although PCSK9 siRNA therapy exhibits powerful effects, the long-term tolerability, efficacy, and safety need to be investigated in more participants. At present, the usage of PCSK9i is restricted to secondary prevention in patients with high CVD risks due to the high expense. In the future, the cost-effective small-molecules with specific PCSK9 inhibitory ability may reduce the manufacturing costs and promote the usage of PCSK9i for primary prevention of ASCVD.

## Glossary

Apo B: apolipoprotein B
ASCVD: atherosclerotic cardiovascular disease
CAD: coronary artery disease
CE: cholesterol ester
CETP: cholesteryl ester transfer protein
CHD: coronary heart disease
CVD: cardiovascular disease
FPLC: fast protein liquid chromatography
GGE: gradient gel electrophoresis
HDL: high-density lipoprotein
HPLC: high-performance liquid chromatography
HPSG: heparan sulfate proteoglycan
IDL: intermediate density lipoprotein
IMT: intima media thickness
LDL: low-density lipoprotein
LDL-C: low-density lipoprotein cholesterol
LDL-P: low-density lipoprotein particle
LDLR: low-density lipoprotein receptor
LOX1: lectin-like ox-LDL receptor
LRP-1: low-density lipoprotein receptor-related protein 1
MTP: microsomal triglyceride transfer protein
NADPH: nicotinamide adenine dinucleotide phosphate
NMR: nuclear magnetic resonance
ox-LDL: oxidized low-density lipoprotein
PCSK9: proprotein convertase subtilsin/kexin type 9
PCSK9i: proprotein convertase subtilsin/kexin type 9 inhibitor
ROS: reactive oxygen species
sdLDL: small dense low-density lipoprotein;
siRNA: small interfering RNA
TG: triglyceride
VLDL: very low-density lipoprotein
WHO: World Health Organization
2D-GE: two-dimensional gel electrophoresis
